# Quantitative Evaluation of Extramural Vascular Invasion of Rectal Cancer by Dynamic Contrast-Enhanced Magnetic Resonance Imaging

**DOI:** 10.1155/2022/3038308

**Published:** 2022-05-31

**Authors:** Zheng Chen, Da Hu, Guannan Ye, Dayong Xu

**Affiliations:** ^1^Department of General Surgery, The First Hospital of Changsha, Changsha 410005, Hunan, China; ^2^Department of Radiology, The First Hospital of Changsha, Changsha 410005, Hunan, China; ^3^Department of Gastroenterology, The First Hospital of Changsha, Changsha 410005, Hunan, China

## Abstract

This study was carried out to explore the preoperative predictive value of dynamic contrast-enhanced magnetic resonance imaging (DCE-MRI) in extramural vascular invasion (EMVI) in patients with rectal cancer. 124 patients with rectal cancer were randomly divided into two groups, with 62 groups in each group. One group used conventional magnetic resonance imaging (MRI) and was recorded as the control group. The other group used DCE-MRI and was recorded as the experimental group. The diagnostic value was evaluated by comparing the MRI quantitative parameters of EMVI positive and EMVI negative patients, as well as the area under the curve (AUC) of the receiver operating characteristic curve (ROC), diagnostic sensitivity, and specificity of the two groups. The results showed that the Ktrans and Ve values of EMVI positive patients in the experimental group and the control group were 1.08 ± 0.97 and 1.03 ± 0.93, and 0.68 ± 0.29 and 0.65 ± 0.31, respectively, which were significantly higher than those in EMVI negative patients (*P* < 0.05). The AUC of EMVI diagnosis in the experimental group and the control group were 0.732 and 0.534 (*P* < 0.05), the sensitivity was 0.913 and 0.765 (*P* < 0.05), and the specificity was 0.798 and 0.756 (*P* > 0.05), respectively. In conclusion, DCE-MRI has a higher diagnostic value than conventional MRI in predicting EMVI in patients with rectal cancer, which was worthy of further clinical promotion.

## 1. Introduction

Extramural vascular invasion (EMVI) refers to the presence of tumor plug attachment or tumor cell infiltration in the blood vessels outside the muscularis propria of the rectal wall [1–4]. It was first reported by Professor Talbot in 1981, and it was pointed out that EMVI could affect the prognosis of rectal cancer patients. For EMVI-positive rectal cancer patients, neoadjuvant chemoradiotherapy followed by surgical treatment can significantly reduce local recurrence and improve prognosis, so it is particularly important to complete accurate preoperative assessment of EMVI [5–8]. Conventional methods for preoperative rectal cancer patients include multi-row spiral CT, endorectal ultrasonography (ERUS), and dynamic contrast-enhanced magnetic resonance imaging (DCE-MRI). Preoperative accurate assessment of rectal cancer depends on the diagnosis and treatment level of radiologists and the development of imaging technology [9–11].

Multi-row spiral CT has poor resolution of pelvic soft tissues, making it difficult to accurately evaluate the depth of tumor invasion into the rectal wall and EMVI [12]. ERUS examination can accurately identify the hierarchical structure of rectal wall based on the principle of acoustic reflection, and has high accuracy in judging the depth of tumor invasion. However, when the tumor is large and late in stage, resulting in intestinal obstruction, the diagnostic accuracy of ultrasound is reduced because it cannot enter the intestinal lumen [13]. DCE-MRI is a noninvasive functional imaging technique that integrates morphologic and hemodynamic changes to reflect the characteristics of tumor microcirculation. Because DCE-MRI can quantify the parameters related to tumor microangiogenesis, perfusion, and permeability, it is widely used in the detection, prediction of EMVI and evaluation of neoadjuvant therapy response, tumor angiogenesis, biological invasiveness, and molecular markers [14]. As far as we know, there are few studies on the evaluation of EMVI of rectal cancer by DCE-MRI. In recent years, some scholars have reported the correlation between MRI evaluation of EMVI of rectal cancer (mrEMVI) and DCE-MRI parameters. The results showed that Kep values of mrEMVI positive patients were significantly lower than those in mrEMVI negative patients, while Ve values were higher than those in mrEMVI negative patients. However, the diagnostic efficacy of DCE-MRI quantitative parameters for EMVI remains unclear [15].

As an emerging area, imaging omics has attracted more and more attention. It mainly conducts quantitative analysis on imaging data and extracts a large number of quantitative features from medical images to provide nonvisual information related to tumor heterogeneity, which can be used for personalized treatment [16]. For rectal cancer, imaging omics is mainly applied to predict the cardiac resynchronization therapy (CRT) response, survival, and other pathological features, such as *T* stage, lymph node metastasis, and peripheral nerve invasion. However, to our knowledge, few studies have reported the use of imaging omics to predict EMVI in rectal cancer [17]. Therefore, in this study, DCE-MRI was used to evaluate EMVI of rectal cancer patients, aiming at exploring the preoperative predictive value of DCE-MRI for EMVI, and providing accurate preoperative risk stratification and individualized treatment for patients.

## 2. Materials and Methods

### 2.1. Research Subjects

In this study, 124 patients with rectal cancer diagnosed in hospital from May 2019 to October 2021 were divided into two groups according to the random number table method, with 62 patients in each group. Conventional MRI scanning was used in the control group, while DCE-MRI scanning was used in the experimental group. All patients and their families had fully understood the situation and signed informed consent, and this study had been approved by the ethics committee of hospital.

Inclusion criteria were as follows: (I) patients who were highly suspected of rectal cancer and confirmed by colonoscopy biopsy and histopathology; (II) patients who underwent the resection surgery within 14 days after DCE-MRI examination and had complete postoperative pathological data; and (III) those with no history of intraperitoneal tumor, and no history of intraperitoneal or anal surgery.

Exclusion criteria: (I) Patients with missing postoperative case data; (II) patients with diseases of the blood system, or patients with coagulation dysfunction or low immune function; (III) patients with serious dysfunction of heart, liver, and kidney; (IV) patients with contraindications for MRI; and (V) patients who did not cooperate with the examination.

### 2.2. Collection of MRI Imaging Data

The MRI scan was performed by a 3.0 T MRI machine. Before the scan, patients were asked to fast for 12 hours to keep the intestine empty. The patient was in a supine position with stable breathing. The scan range was from the lowest point of the symphysis pubis to the iliac crest line on both sides. The scan was carried out according to the DCE-MRI scan sequence specification for rectal cancer. (1) Coronal and sagittal T2-weighted imaging (T2WI) sequences: Fast Spin Echo (FSE) sequence was used for scanning. (2) Axial T2WI sequence: the tumor location was first determined by sagittal T2WI, and then horizontal FSE scan was performed along the long axis of the rectum perpendicular to the tumor. (3) Diffusion weighted imaging (DWI) sequence: tumor location was determined by sagittal T2WI, and horizontal scanning was performed using single excitation spin echo-planar imaging (EPI) technology. (4) Dynamically enhanced T1-weighted imaging (T1WI) scan sequence: according to the patient's body weight, the injection volume of the required contrast agent was calculated at a dose of 0.2 mL/kg, and the enhanced contrast agent-Gadolinium diamine injection was injected with a high-pressure syringe at a constant rate.  Table 1 shows the specific scan parameters of each scan sequence.

### 2.3. DCE-MRI Image Processing

The imaging evaluation of each patient was agreed upon by two abdominal radiologists with more than 5 years of experience in pelvic MRI diagnosis, and the physician who evaluated the images was unaware of relevant laboratory and pathological findings. Pharmacokinetic analysis of DCE-MRI parameters was performed. The images were independently evaluated by two radiologists and regions of interest (ROI) were delineated layer by layer. Meanwhile, it should be noted to keep away from visible blood vessels, peripheral fat, necrosis or hemorrhage, and intestinal lumen contents should be avoided as far as possible. Then, the ROI of all layers should be fused to obtain the total ROI of the tumor, and the quantitative parameters below were recorded: volume transfer constant (Ktrans), rate constant (Kep), volume fraction of extravascular extracellular space (Ve), and volume fraction of plasma (Vp). Semiquantitative parameters: initial area under curve (iAUC), time to peak (TTP), rising slope (Max Slope), and maximum concentration of contrast agent (Max Conc).

The imaging evaluation of EMVI was conducted according to the MRI evaluation EMVI scoring system proposed by Leithner et al. [18]. 0: The tumor is non-nodular, infiltrating into the muscular layer, and there are no blood vessels outside the intestinal wall around the tumor; 1: The tumor is nodular, infiltrating into the muscle layer or having tiny blood vessels outside the intestinal wall, but not around the tumor; 2: there are blood vessels outside the intestinal wall around the tumor, but the size of the blood vessels is normal and there is no clear tumor signal in the blood vessels; 3: there was a moderate intensity signal in the blood vessels around the tumor, but the outline and diameter of the blood vessels only changed slightly; and 4: tumor signal appears in the blood vessels around the tumor, and the outline of the blood vessels is obviously irregular or the blood vessels are nodular. 0∼2 is EMVI negative, and 3∼4 is EMVI positive.

### 2.4. Statistical Methods

The Shapiro–Wilk test is performed to determine whether the variables are normally distributed. The *T* test or Mann–Whitney *U* test is used for continuous variables, and Chi-square test or Fisher's exact test is used for categorical variables. Univariate and multivariate logistic regression analyses are carried out to determine independent predictors of EMVI. The receiver operating curve (ROC) is drawn and area under the curve (AUC) is calculated to evaluate the diagnostic efficacy of each imaging omics model, and internal verification is carried out in an independent verification set. The diagnostic efficacy of clinical, quantitative, and radiomic models are evaluated based on AUC, sensitivity, and specificity. Intraclass correlation coefficient (ICC) and 95% confidence interval (CI) are calculated to analyze the interobserver consistency of DCE-MRI quantitative perfusion parameters measured by two radiologists. *P* < 0.05 indicates that the difference is statistically significant.

## 3. Results

### 3.1. Clinical Pathological and Imaging Data of the Patients

**Figure 1:**
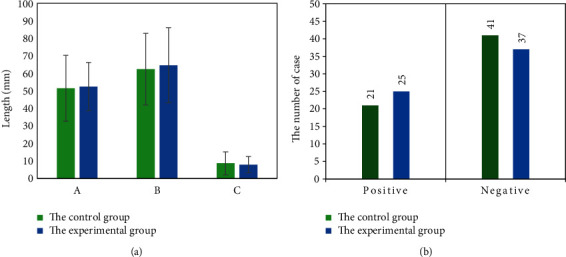
Clinical pathological characteristics of the two groups. In Figure (a), A represents the diameter of the tumor, B represents the distance between the lower margin of the tumor and the anal margin, and C represents the depth of tumor invasion. Figure (b) shows the number of EMVI cases.

**Figure 2:**
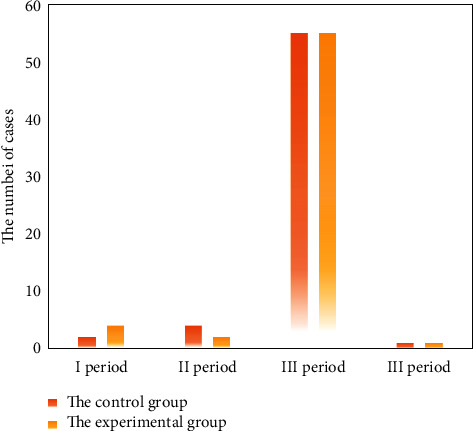
TNM staging in both groups.

**Figure 3:**
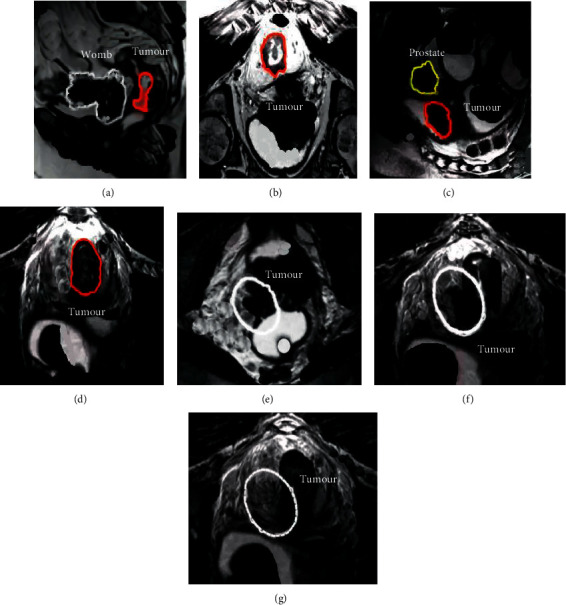
MRI images of a patient with rectal cancer. In Figure 3, arrows and dotted lines indicate cancerous sites. (a, c) show sagittal T2-weighted images. (b, d) Axial T2-weighted images. (e) Coronal T2-weighted image, and (f, g) show continuous axial MRI images. (e–g) Condition of a positive EMVI case. Under normal circumstances, the MRI images of large vessels outside the rectal wall should show a creeping distribution, while the blood vessels in (e–g) show signal loss on T2WI images due to blood flow in the vessels, also known as emptying phenomenon. When the external vascular lumen of rectum is enlarged, the irregular contour and the phenomenon of empty flow disappear and are replaced by tumor signal, namely, EMVI.

The mean age of patients in the experimental group was (62.1 ± 11.4) years old and that in the control group was (61.9 ± 10.9) years old. In the control group, there were 21 positive EMVI cases and 41 negative EMVI cases. The numbers were 25 and 37 in the experimental group. There was no significant difference in age, tumor diameter, number of EMVI cases, and TNM stage between the two groups (*P* > 0.05), which was comparable. Figures 1–3 show the clinical pathological characteristics of patients in the two groups, as well as the MRI images.

### 3.2. Comparison of DCE-MRI Parameters between the EMVI Positive Group and Negative Group

**Figure 4:**
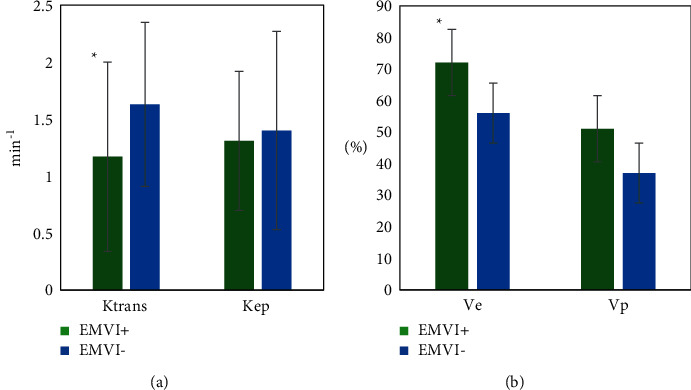
DCE-MRI parameters of the EMVI positive group and negative group. (a) Ktrans and Kep; (b) Ve and Vp. ^∗^means *P* < 0.05, the difference is statistically significant.

**Figure 5:**
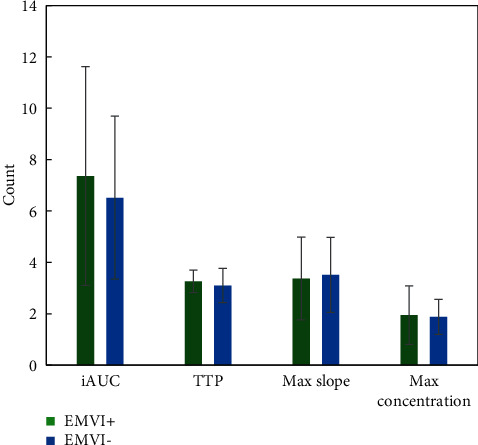
DCE-MRI parameters of the EMVI positive group and negative group.

The consistency between two clinicians was good for the measurement of quantitative and semiquantitative parameters of DCE-MRI. The intragroup correlation coefficients of Ktrans, Kep, Ve, Vp, iAUC, TTP, Max Slope, and Max Conc were 0.78, 0.83, 0.86, 0.85, 0.88. 0.81, 0.75, and 0.73, respectively. Figures 4 and 5 show DCE-MRI parameters in the EMVI positive group and the EMVI negative group. Ktrans and Ve values in EMVI positive group were significantly higher than those in EMVI negative group, *P* < 0.05. There was no significant difference in Kep, Vp, and semiquantitative parameters between the EMVI positive group and EMVI negative group, *P* > 0.05.

**Figure 6:**
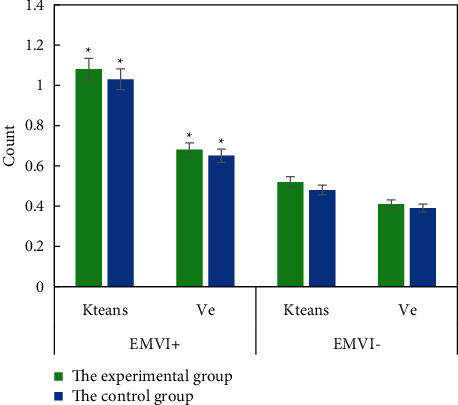
Ktrans and Ve values of EMVI positive and negative patients in the two groups. ^∗^means the difference is statistically significant, *P* < 0.05.

The Ktrans value and Ve value of EMVI-positive patients in the experimental group and control group were 1.08 ± 0.97 and 1.03 ± 0.93, 0.68 ± 0.29 and 0.65 ± 0.31, respectively, which were significantly higher than those of EMVI-negative patients, *P* < 0.05, and the difference was statistically significant, as shown in Figure 6.

### 3.3. DCE-MRI Parameters of the Two Groups

**Figure 7:**
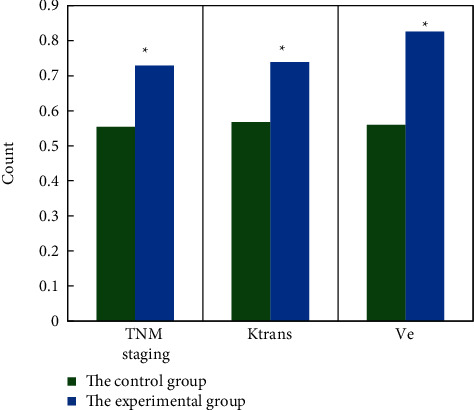
Diagnostic performance of different parameters in two groups. ^∗^means the difference is statistically significant, *P* < 0.05.

In this study, the diagnostic efficacy of various parameters for EMVI was evaluated by drawing ROC and calculating AUC. It was found that the DCE-MRI parameter performance (TNM staging, Ktrans, Ve) of the experimental group was significantly higher than that of the control group (*P* < 0.05), and the difference was statistically significant, as shown in Figure 7.

**Figure 8:**
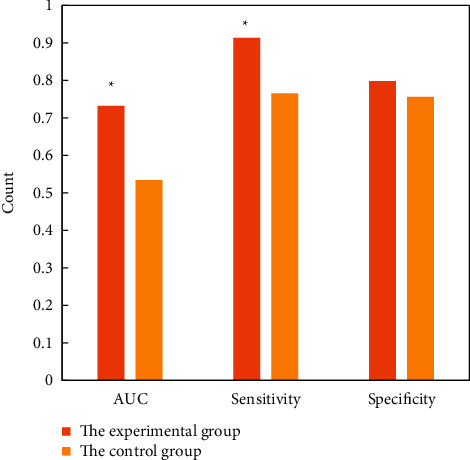
Comparison of AUC, sensitivity, and specificity of EMVI diagnosis between the two groups. ^∗^means the difference is statistically significant, *P* < 0.05.

The AUC of EMVI diagnosis in the experimental group and the control group was 0.732 and 0.534, and the diagnostic sensitivity was 0.913 and 0.765, respectively. The values of the experimental group were significantly higher than the control group, *P* < 0.05. The specificity of diagnosis was 0.798 and 0.756, respectively, with no significant difference between the two groups (*P* > 0.05), as shown in Figure 8.

## 4. Discussion

According to statistics, EMVI can occur in about 1/3 of rectal cancer patients, and EMVI is very important for the prognosis of patients and the choice of treatment plan. The eighth edition of *American Joint Council on Cancer* (AJCC) include it as an independent adverse prognostic factor and as class I evidence. In addition, EMVI has been recommended as an imaging marker to predict the efficacy of neoadjuvant chemotherapy [19, 20]. Previous studies have shown that risk factors for EMVI include large tumor size and high *T* and *N* stages. In this study, the TNM stage was confirmed to be an independent predictor of EMVI, which was partially consistent with previous studies [21]. However, it was found that tumor size and TNM stage were not correlated with EMVI. In terms of tumor staging, this result may be due to selection bias, and the ROI of the tumor is manually delineated, which may be influenced by the individual factors of the delineator. Therefore, these factors cannot be used in preoperative decision making alone. Chen et al. showed that lymphovascular invasion (LVI) of rectal cancer could be evaluated preoperatively by measuring the total tumor volume on DWI and T2W images, and its AUC value was 0.899 and 0.877, respectively. However, volume measurement needs a lot of time, and cannot fully reflect the nature of EMVI, and the result remains to be verified [22]. Sun et al. attempted to explore the value of DWI as a potential quantitative method in the evaluation of rectal cancer EMVI, but the diagnostic efficacy was not significantly improved when DWI was added to detect EMVI [23].

In this study, the correlation between DCE-MRI parameters and EMVI in rectal cancer was analyzed, and only Ktrans and Ve values were significantly different between EMVI positive and EMVI-negative groups. The results showed that Ktrans and Ve values in EMVI positive group were significantly higher than those in negative group, and Ktrans and Ve values were positively correlated with EMVI, suggesting that these two parameters may be closely related to vascular invasion of tumor. Ve represents the volume fraction of extracellular extravascular space (EES). In the process of tumor progression, tumor cells secrete vascular endothelial factor, which increases the permeability of tumor vessels, and the function of cell-cell adhesion molecules is lost, leading to the expansion of cell space and EES. Therefore, it can be reasonably explained that the Ve value of EMVI positive group is significantly higher than that of EMVI negative group. This result was consistent with that of Leijssen et al. who reported increased Ve values in mrEMVI positive patients [24]. In terms of Ktrans, our findings are consistent with a recent study showing that LVI is associated with a high Ktrans value in rectal cancer patients [25].

Chinese Society of Clinical Oncology (CSCO) guidelines for the diagnosis and treatment of colorectal cancer recommend the use of rectal MRI to determine EMVI [26]. Other research results showed that the accuracy, sensitivity, specificity, positive predictive value, and negative predictive value of MRI detection of EMVI were 81%, 62%, 88%, 0.67, and 0.86, respectively [27]. Another study showed that the sensitivity and specificity of MRI for EMVI recognition were 28.2%–62.0% and 88.0%–94.0% [28]. In this study, DCE-MRI was used for preoperative prediction of EMVI in rectal cancer patients. It was found that the AUC of EMVI diagnosis in the experimental group and the control group was 0.732 and 0.534, respectively, and the diagnostic sensitivity was 0.913 and 0.765, respectively. Obviously, the values in the experimental group were significantly higher than the control group, *P* < 0.05. The specificity of diagnosis was 0.798 and 0.756, respectively, with no significant difference between the two groups (*P* > 0.05). Above, DCE-MRI is feasible and efficient and can assist imaging doctors in imaging diagnosis.

## 5. Conclusion

In this study, DCE-MRI was used for preoperative prediction of EMVI in patients with rectal cancer. DCE-MRI has a higher diagnostic value in predicting EMVI in rectal cancer patients than conventional MRI, which is worthy of further clinical promotion. However, in this study, tumor images below the level of levator ani muscle and above the peritoneal recursion are not included, so its applicability is limited. Besides, the sample size of this study is small, and an expanded sample size is necessary to strengthen the findings of the study. In a word, this study can provide theoretical basis for preoperative risk stratification and individualized treatment of patients.

## Figures and Tables

**Table 1:** MRI scan sequence parameters.

	Sagittal T2WI	Coronal FS T2WI	Axial FS T2WI	Axial DWI
Repetition time (ms)	3000 ms	3000 ms	3000 ms	4000 ms
Echo time	(70–110) ms	(70–110) ms	(70–110) ms	(70–110) ms
Layer space	1.0 mm	2.0 mm	1.5 mm	1.5 mm
Layer thickness	5.0 mm	6.0 mm	5.0 mm	5.0 mm
Matrix	350 × 256	350 × 186	350 × 186	120 × 150
Field of view	(33–45) cm	(37–45) cm	(37–45) cm	(37–53) cm
Number of excitations	2	3	4	5

## Data Availability

The data used to support the findings of this study are available from the corresponding author upon request.
